# Protein Hydrolysates’ Absorption Characteristics in the Dynamic Small Intestine In Vivo

**DOI:** 10.3390/molecules23071591

**Published:** 2018-06-29

**Authors:** Yuanqing He, Lingling Shen, Chaoyue Ma, Min Chen, Ye Pan, Lijing Yin, Jie Zhou, Xiaochun Lei, Qian Ren, Yuqing Duan, Haihui Zhang, Haile Ma

**Affiliations:** 1 College of Food Science and Biological Engineering, Jiangsu University, Zhenjiang 212013, China; slingling10@163.com (L.S.); machaoyue1992@163.com (C.M.); 18753364685@163.com (M.C.); panye@ujs.edu.cn (Y.P.); dyq101@ujs.edu.c (Y.D.); zhanghh@ujs.edu.cn (H.Z.); mhl@ujs.edu.cn (H.M.); 2 The Laboratory Animal Research Center, Jiangsu University, Zhenjiang 212013, China; ljyin@ujs.edu.cn (L.Y.); jiera_qd@163.com (J.Z.); sandra1116@126.com (X.L.); renqian@ujs.edu.cn (Q.R.)

**Keywords:** small intestine model, protein hydrolysate absorption, in vivo

## Abstract

*Background*: Dietary proteins are known for their wide range of nutritional, functional and biological properties. Although the total amount of proteins may be obtained from mixtures, its “availability” for absorption in the gut is in many cases quite uncertain or even varies for the same food depending on processing conditions, the presence of other components, and so on. *Methods*: To obtain accurate protein hydrolysate absorption data, we have developed a small intestine model (SIM) to test them. *Results*: The results indicated that the protein hydrolysates were absorbed rapidly during the first 15 min, and then decreased to 90 min, then they were absorbed again from 90 min to the endpoint. The protein absorption was also affected by the protein processing method used. The Enzyme + Ultrasound (EU) processing method group had a higher absorption rate than the Enzyme (E) processing method group, and the absorption of the Enzyme + Artificial gastric juice processing method (EH) and Enzyme + Ultrasound + Artificial gastric juice processing method (EUH) groups was reduced compared to the E group alone. The amino acid analysis results showed that the amino acids were reduced and absorbed by our SIM in almost all groups except for cysteine and methionine. In general, the Pearson relation value of the amino acid contents between before SIM and after SIM was 0.887, which indicated that single amino acid absorption was mainly related to its content in the whole amino acids. The single amino acid absorption ratio among different groups also displayed differences, which ranged from 31% to 46% (E group from 39% to 42%; EU group from 40% to 47%; EH group from 31% to 39%; EUH group from 35% to 41%). Conclusions: The protein hydrolysates’ varied from startpoint to endpoint, and the protein absorption was affected by processing method.

## 1. Introduction

Dietary proteins are known for their wide range of nutritional, functional and biological properties [1]. According to the data of the Institute of Medicine (IOM) of the World Health Organization/Food and Agriculture Organization/United Nations University (WHO/FAO/UNU), the Recommended Dietary Allowance (RDA) of protein for a healthy adult with minimal physical activity was 0.8 g protein per kg body weight (BW) per day. During the last 20 years, bioactive peptides or protein hydrolysates have become of increasing scientific and commercial interest owing to their ability to positively affect the major body systems, notably, the cardiovascular, digestive, endocrine, immune and nervous systems, while minimizing the risks of chronic disease development [1,2,3,4]. However, when studying the effect of bioactive peptide compounds in our organism, it is important to verify the rate, extent, and location of protein hydrolysis within the human gastrointestinal tract (GIT) and ensure the active form reaches the target organ(s). For this purpose, stability to digestion has to be assessed and, if it is absorbed, it is also important to evaluate the distribution, metabolism, and excretion [2,5].

The digestive tract is the system of organs used to extract energy and nutrients from foods and expel waste. It is notable that the four processes act strictly in series [6]. In the mouth the food is comminuted by a mixture of cutting and grinding by the teeth and squeezing by the tongue and cheeks, brought to approximately body temperature, mixed well with saliva and tasted. In the stomach the food is accumulated and digested. The cells in the gastric wall secrete large amounts of hydrochloric acid to form a strongly acidic environment in stomach. Then two enzymes are secreted into the stomach that catalyse the breakdown of proteins (pepsin) and fats (gastric lipase). In the small intestine the food (proteins, fats and carbohydrates) is hydrolyzed by hydrolytic enzymes and absorbed through the large surface area of the ileum and jejunum. In the large intestine the residual food components that have not already been digested are fermented by microorganisms. Some products are absorbed and the others are egested [6,7,8]. On the other hand, the digestion and absorption of food in the human gastrointestinal tract (GIT) system is a complex process including physical, chemical and biological process, and little is known about its biological process. Bioactive peptide or protein hydrolysates absorption could occur in the GIT regulation after they enter the body, and then regulate the whole hormone system resulting in induced satiety, delayed gastric emptying, increased gastrointestinal transit time, and changed food intake, affecting digestion and absorption again [9].

In recent years in vitro methods of simulating digestion processes are widely used to study the gastro-intestinal behavior of food [10]. Sophisticated semi-soft and soft gastric models have been developed, including the TNO gastric model (TIM-1), the human gastric simulator (HGS), the dynamic gastric model (DGM), the human gastric digestion simulator (GDS), and in vitro mechanical gastric system (IMGS) [11,12,13,14,15,16]. In vitro methods have the advantage of being more rapid, less expensive, and less labour intensive, and do not have ethical restrictions. Furthermore, they allow a relatively large number of samples to be measured in parallel for screening purposes [10]. Among these models, the TNO in vitro gastrointestinal model (TIM-1 system) has been applied for the digestion and availability studies of the absorption of various nutrients in comparison with in vivo studies, especially for the absorption of iron in foods [17,18,19,20]. However, it still cannot be applied to analyze the absorption and bioavailability of protein complex when considering the biological effects. There are three absorption models, the Caco-2 cells, Ussing chamber with ex vivo different rat intestinal segments, and the in situ intestinal perfusion model in rat often be used in this research [2,21]. In this study, we developed an in vivo small intestinal absorption model to accurately obtain the absorption characteristics of food protein hydrolysates.

## 2. Results

### 2.1. Food Running Speed

As we all knew, the food running speed was affected by many factors such as the food components, food concentrations, food pH, food ion components, etc. These factors will vary from product to product. Therefore, before protein absorption changes are tested, we should first confirm the in vivo food running speed in the intestine. The food running speed for oat protein hydrolysates is 0.15 mL/min, which was also used as the speed of the peristaltic pump.

### 2.2. Protein Absorption Change with the Time

**Figure 1:**
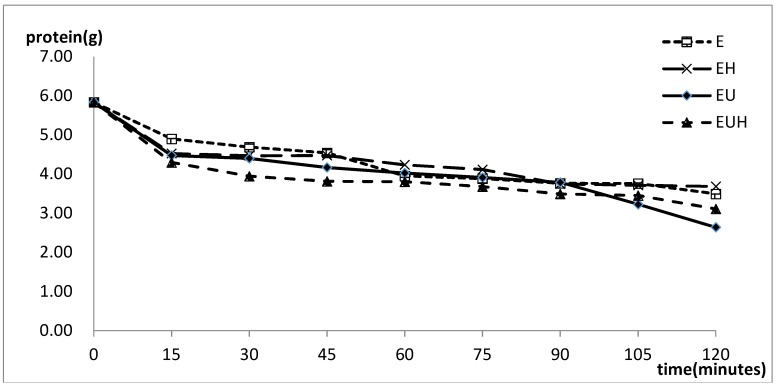
The protein content changes in different groups with the time.

The protein content changes with time are shown in Figure 1. The results showed that the protein content decreased with time in all three groups and the protein hydrolysates were absorbed through the SIM model. At the same time, we interestingly found that the protein absorption nearly reacha common value at 60 min despite the processing method used, and the protein absorption changed little from 60 min to 90 min. After 90 min, the protein absorption in the different groups showed differences again. We thought that the intestine absorption reached saturation when the food was absorbed up to 60 min, then the absorption started again at 90 min when the absorbed proteins were taken away by the blood. 

**Figure 2:**
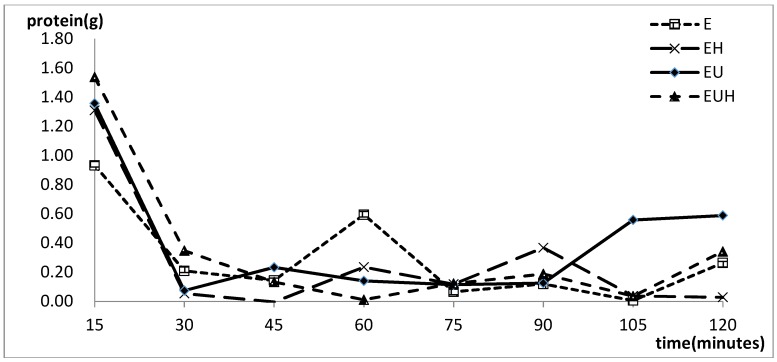
The absorption changes in different groups with time.

Figure 2 shows the absorption changes in the different groups with time. The results indicated that the proteins were absorbed rapidly during the first 15 min, and then the absorption started to differentiate among the groups. E group absorption was reduced from 15 to 45 min and increased from 45 to 60 min, then reduced from 60 to 105 min, finally increasing to the end point. The EU group and EUH group had an obvious stage with almost stopped absorption from 15 to 90 min, which revealed that in this period less absorption happened. The main absorption period was the first 15 min and the last 15 min.

### 2.3. Total Protein Absorption Difference

**Figure 3:**
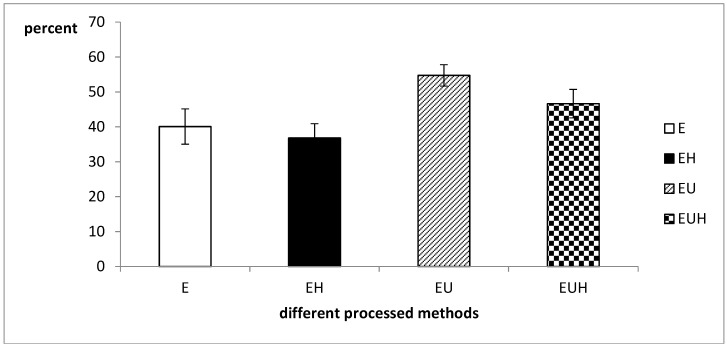
The absorption difference of total protein in different groups.

Protein hydrolysate absorption after different processing methods was detected through SIM model. The absorption results are shown in Figure 3. The results indicated that the absorption ratio ranged from 45 percent to 50 percent, and EU group had a higher absorption rate than the other groups. It is well known that the ultrasound pretreatment step will break up the complex structure of the oats and display the protein surface structures, which will increase the efficiency of the enzyme, and then increase the content of small molecule proteins, peptides and amino acids, which could be absorbed by the intestine easily. On the other hand, in order to confirm whether the gastric digestion could increase the intestine absorption, we designed the EH and EUH group with a simulated gastric fluid treatment after the E group and EU group processing steps. The results indicated that there is no significant difference in absorption between E and EH groups or between the EU and EUH groups. These results indicated that the simulated gastric fluid could not increase the absorption of food proteins. We speculate that the processing methods fully replaced the enzyme treatment step, and the simulated gastric fluid step was just a repeated step.

### 2.4. Amino Acid Absorption Difference

**Figure 4:**
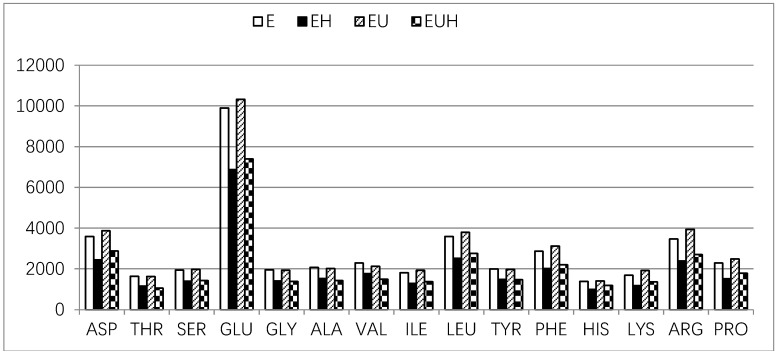
The amino acid absorption of oat protein hydrolysates in different groups.

**Figure 5:**
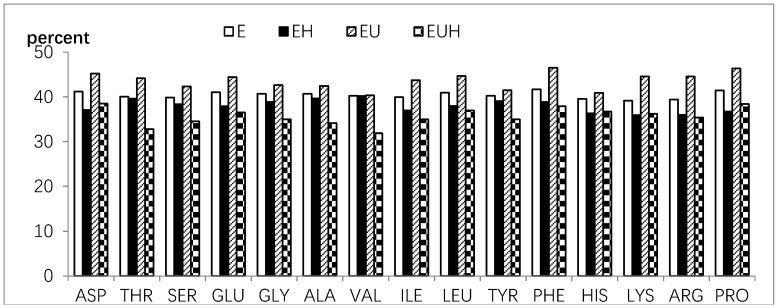
The single amino acid absorption ratio of oat protein hydrolysates in different groups.

The amino acid composition was tested before SIM and after SIM, and the amino acid absorption was calculated as the difference between before SIM and after SIM. The results are shown in Figure 4 and Figure 5. Figure 4 shows that all amino acids were reduced and absorbed in the E group and EH group, and all amino acids, except cysteine and methionine, were reduced and absorbed in the EU and EUH groups after SIM. As we all know cysteine and methionine could be changed and undergo complex transformations in vivo depending on the body requirements. 

In general, the Pearson relation value between before SIM and after SIM of the amino acid contents was 0.887 (E group = 0.997, EU group = 0.973, EH group = 0.826, EUH group = 0.901; *p* < 0.01), which indicated that single amino acid absorption was mostly related to its content in whole amino acids. On the other side, the single amino acid absorption ratio among different groups also showed differences, and the results are shown in Figure 5. The amino acid absorption ratio ranged from 31% to 46% (E group from 39% to 42%; EU group from 40% to 47%; EH group from 31% to 39%; EUH group from 35% to 41%), which indicated that the amino acid absorption is selective and has its own rules. The results also indicated that, compared to EH and EUH group, the E group and EU group increased the single amino acid absorption ratio. These results implied that the processing method has an effect on the single amino acid absorption.

## 3. Discussion

Protein is an important macronutrient as a source of essential amino acids and energy. In addition to basic nutrition, some food proteins can provide extra health benefits through the release of bioactive peptides encrypted in their sequences. In recent years, research on bioactive peptides or protein complexes including bioactive peptides for the production of value-added food ingredients have attracted great attention among food scientists worldwide [22]. Bioactive peptides or protein hydrolyses can be obtained from different food protein sources such as milk, whey, egg, marine fish species, soybean, rice, peanut, chickpea, amaranth, corn, and algae, which have been shown to display a wide range of physiological functions including antihypertensive, antioxidative, opioid agonistic, immunomodulatory, antimicrobial, prebiotic, mineral binding, antithrombotic and hypocholesterolemic effects [22,23,24,25]. Meanwhile, owing to the structural characteristics of proteins and their amino acid residues positioned on the N-terminal and C-terminal, bioactive peptide fragments might exhibit natural bioresistance or predictable “shelf-life” under gastrointestinal tract conditions. This natural resistance is important for biopeptides to ensure their maximum bioavailability [26]. Therefore, a suitable in vivo model must be developed for testing the protein absorption and bioavailability of bioactive peptides. 

The intestine is the most important organ for food absorption. The majority (80%) of nutrient absorption occurs in the small intestine, where chyme enters from the stomach and it is further broken down into nutrients [7]. In 2010 a study on a simulated model of the small intestine was reported [6]. The authors constructed a small intestine model (SIM) to analyze the food absorption from the food transfer process and food viscosity in the small intestine. This research and design have made tremendous progress in simulating GIT action on the food characteristics such as food pH, food viscosity, food chemical change, enzymes, etc. The model we developed in this research could be regarded as a supplemental model that will further explain the absorption of food in the small intestine in vivo, especially to help analyze the absorption of protein hydrolysates or bioactive peptides. In this SIM model, we tested the food running speed in the intestine, the protein absorption with time, the protein content changes with time, and the total protein and amino acid absorption after applying different processing methods. The indexes were used to test the feasibility of the SIM model, and the different processing methods were used to test the accuracy of the SIM model. The results indicated that the model is effective and offers some interesting data. The results of protein content absorption changes indicated that the proteins were absorbed rapidly during the first 15 min, and then stopped up to about 90 min. Finally, the proteins were absorbed again from 90 min to the endpoint. These results implicated that the processes of digestion, secretion of digestive enzymes and then absorption are actually regulated by the neuro-endocrine system [27]. At the same time, the total protein and amino acid absorption showed that the proteins and the different amino acid were absorbed by the SIM model and differences existed. All these results revealed that the SIM model has enough efficiency and accuracy for food protein analysis.

On the other hand, as we all know, the human body is permanently colonized by microbes on virtually all environmentally exposed surfaces, the majority of which reside within the gastrointestinal (GI) tract [28]. In recent years, researchers have found that the intestinal microbiota influence neurodevelopment, mental health, modulate behavior, cancer immunotherapy and contribute to neurological disorders through microbiota-brain-gut axis [28,29,30,31,32]. The microbiota-brain-gut axis will regulate the food digestion and absorption including protein, fat fiber and the other ingredients [33,34]. This implies that the in vivo model is more accurate than the in vitro model when considering the interactions between food and microbiota. Furthermore, with the development of research on the GIT and microbiota, more and more gut microorganisms will be identified and their function illuminated, and the mechanism of absorption and transformation of protein will become clearer in the future.

## 4. Materials and Methods

### 4.1. Protein Preparation

Oat protein was extracted and isolated following the method of alkali extraction and acid precipitation. In brief oat was ground using an electric grinder and sieved through a 60 mm mesh. The oat flour was dissolved in water with a solid-liquid ratio of 1:9, the pH was adjusted to 10 with 1 mol/L NaOH and reacted for 90 min at 50 °C. The resultant suspensions were centrifuged at 4000× *g* for 15 min. The supernatants were adjusted to pH 4 with 1 mol/L hydrochloric acid to precipitate the proteins. Then the proteins were centrifuged at 4000× *g* for 15 min to collect the protein precipitates. Finally, oat protein powder is obtained by a freeze drying method.

### 4.2. Preparation of Protein Hydrolysates

Oat protein hydrolysates were prepared by four kinds of processing method. For group 1 (E group) the following steps were used: Alcalase 2.4 L FG was used to hydrolyse the oat protein. Optimum variables were: oat protein concentration of 8%, enzyme content, enzymolysis pH, temperature and time of 4000 U/g, 9.5, 55 °C and 90 min, respectively. The enzyme hydrolysate was inactivated for 15 min at 85 °C and centrifuged at 4000× *g* for 15 min after rapid cooling, and the supernatant was collected. The group 2 (EH group) treatment was as follows: after the Group 1 procedure was finished, the sample was mixed with artificial gastric juice (dilute hydrochloric acid 1.64 mL and pepsin 1 g dissolved in 100 mL deionized water). The Group 3 (EU group) treatment was as follows: before the hydrolysis step ultrasound (60 W/L power and 30 min) was applied, the other steps were similar to those of Group 1. For Group 4 (EUH group), after the Group 3 steps were finished, the sample was mixed with artificial gastric juice (dilute hydrochloric acid 1.64 mL and pepsin 1 g dissolved in 100 mL deionized water). All these groups will form protein hydrolysates after finishing the corresponding processing procedure.

### 4.3. Small Intestinal Model (SIM)

**Figure 6:**
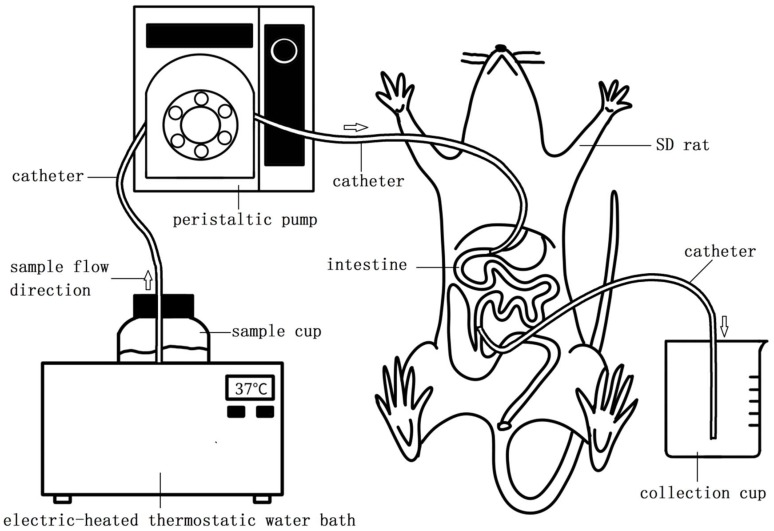
Schematic of the experimental SIM: 1-samle recipient; 2-peristaltic pump; 3-intestinal (from cardia of stomach to distal ileum); 4-SD rat; 5-recycling recipient; 6-sample flow direction; 7-catheter. A surgical opening was formed by surgical scissor at cardia of stomach and distal ileum respectively, and then inserted and tied a catheter with 2.4 mm inner diameter. The other terminal of the catheter was linked to sample cup through the peristaltic pump and recycling recipient respectively. The speed of the peristaltic pump is 0.15 mL/min for the oat protein hydrolysates in this experiment.

The SIM was developed in our research according to the in vivo medicine absorption detection method. The model (a schematic shown in Figure 6) simulated the food running process in the intestine. Sprague-Dawley (SD) rats weighing 180–200 g were obtained from the Laboratory Animal Research Center of Jiangsu University. All animals were housed in groups under a 12-h light/dark cycle (lights on at 7:00 a.m., lights off at 7:00 p.m.) under controlled temperature (22 ± 2) °C and humidity (50 ± 10)%, and fed with standard diet and water ad libitum. They were allowed three days to adapt to the environment, and then changed to food deprivation for 24 h before the experiments started. The rats were narcotized by sodium pentobarbital and the abdomen opened along the medioventral line, exposing the intestines. A surgical opening was formed using surgical scissors on the cardia of the stomach and distal ileum, respectively, and then a catheter with 2.4 mm inner diameter was inserted and tied. The other terminal of the catheter was linked to a sample cup through a peristaltic pump and collection cup, respectively. The flow direction of samples is shown in Figure 6. The food was prepared and put in the sample recipient, from where it entered the intestine and was passed through the intestine by the peristaltic pump, and after these processes the food was gathered by the recycling recipient. The absorption of the food protein was calculated by comparing the protein differences between the sample food and the recycled food. The protocol for the study was reviewed and approved by the Animal Use and Care Committees of Jiangsu University.

### 4.4. In Vivo Intestinal Food Running Speed Measurement 

Sprague-Dawley (SD) rats weighing 180–200 g were obtained from the Laboratory Animal Research Center of Jiangsu University. All animals were fed in groups under the same circumstances. After adaption for three days, rats were subjected to food deprivation for 24 h, and then they were fed colored food samples (without toxic effects) by intragastric administration. After 20 min, the food running speed was calculated by the food running distance during this period.

### 4.5. Protein Absorption Change with the Time

All groups were tested in the SIM model and samples collected in the recycling recipient at 15, 30, 45, 60, 75, 90, 105, 120 min, and a 0 min sample was collected in the sample recipient. The protein content of recycled samples was calculated by the Kjeldahl method.

### 4.6. Total Protein and Amino Acid Absorption

In each group, samples were collected at the start and end point in the SIM. The total protein absorption was tested at the two time points by the Kjeldahl method, and the amino acid absorption was tested by an amino acid analyzer (Sykam S-433, Berlin, Germany).

### 4.7. Statistical Analysis

Statistical analysis of data was performed with the SPSS statistical software (version: 19.0, IBM, Armonk, NY, USA). Differences were considered statistically significant if the *p* value was <0.05.

## 5. Conclusions

Protein hydrolysates’ absorption has specific characteristics in vivo. Amino acid absorption is not only related to its content in whole amino acids but also follows certain rules.
